# An Enquiry into the Pathological Importance of Ulceration of the Os Uteri

**Published:** 1856-01

**Authors:** 


*An Enquiry into the Pathological Importance of Ulceration of the Os Uteri.* By Chas. West, M. D. &c., &c. Phila—Blanchard & Lea, 1854.

To those who are at all acquainted with any of the published writings of this author, his name alone will lead them to read any work to which it is attached. His work on the diseases of children is a model of its kind—showing great powers of observation, clear discrimination, and of unfailing practical suggestion.

This little work, consisting of two Lectures, (the Croonain Lectures for 1854,) enters as thoroughly as could be done in so little space, into the proposed subject, and does much to enlighten upon the disorders of the uterus, too many of which are blindly referred to ulcerations of the Os and Cervix. The argument is enforced by statistical illustrations, and no one can peruse the work without being convinced, as the author remarks, "that there is here, no more than elsewhere, any royal road to knowledge, and it remains for us only to make out, as best we may, with what varied states of the general health, or of the sexual system, the various signs of uterine disease are connected; to learn how, in different cases, we may surely distinguish, or even shrewdly guess their import." We trust all will read the little book. It is very neatly gotten up by the publishers.

